# Patterns of *Limnohabitans* Microdiversity across a Large Set of Freshwater Habitats as Revealed by Reverse Line Blot Hybridization

**DOI:** 10.1371/journal.pone.0058527

**Published:** 2013-03-12

**Authors:** Jan Jezbera, Jitka Jezberová, Vojtěch Kasalický, Karel Šimek, Martin W. Hahn

**Affiliations:** 1 Biology Centre of the ASCR, v.v.i., Institute of Hydrobiology, České Budějovice, Czech Republic; 2 University of Innsbruck, Research Institute for Limnology, Mondsee, Austria; J. Craig Venter Institute, United States of America

## Abstract

Among abundant freshwater *Betaproteobacteria*, only few groups are considered to be of central ecological importance. One of them is the well-studied genus *Limnohabitans* and mainly its R-BT subcluster, investigated previously mainly by fluorescence *in situ* hybridization methods. We designed, based on sequences from a large *Limnohabitans* culture collection, 18 RLBH (Reverse Line Blot Hybridization) probes specific for different groups within the genus *Limnohabitans* by targeting diagnostic sequences on their 16 S–23 S rRNA ITS regions. The developed probes covered in sum 92% of the available isolates. This set of probes was applied to environmental DNA originating from 161 different European standing freshwater habitats to reveal the microdiversity (intra-genus) patterns of the *Limnohabitans* genus along a pH gradient. Investigated habitats differed in various physicochemical parameters, and represented a very broad range of standing freshwater habitats. The *Limnohabitans* microdiversity, assessed as number of RLBH-defined groups detected, increased significantly along the gradient of rising pH of habitats. 14 out of 18 probes returned detection signals that allowed predictions on the distribution of distinct *Limnohabitans* groups. Most probe-defined *Limnohabitans* groups showed preferences for alkaline habitats, one for acidic, and some seemed to lack preferences. Complete niche-separation was indicated for some of the probe-targeted groups. Moreover, bimodal distributions observed for some groups of *Limnohabitans*, suggested further niche separation between genotypes within the same probe-defined group. Statistical analyses suggested that different environmental parameters such as pH, conductivity, oxygen and altitude influenced the distribution of distinct groups. The results of our study do not support the hypothesis that the wide ecological distribution of *Limnohabitans* bacteria in standing freshwater habitats results from generalist adaptations of these bacteria. Instead, our observations suggest that the genus *Limnohabitans*, as well as its R-BT subgroup, represent ecologically heterogeneous taxa, which underwent pronounced ecological diversification.

## Introduction

*Betaproteobacteria* usually represent a major fraction and frequently even the numerically dominating fraction of bacterioplankton in lentic (standing) freshwater systems [1], [2]. Eighteen distinct lineages of *Betaproteobacteria* have been identified as typical constituents of freshwater bacterioplankton [1], however, only few lineages were found to be of ecological as well as numerical importance in lentic freshwater habitats [1], [2]. These lineages are represented by two genera; *Polynucleobacer* and *Limnohabitans* [2]. The recently described genus *Limnohabitans* (*Comamonadaceae*, [3]) currently comprises four described species [3]–[5] in four tribes (Lhab-A1 to Lhab-A4) [1]. Both betaproteobacterial taxa have been described as abundant parts of freshwaters bacterioplankton, responding fast to changing environmental conditions, frequently forming together a vast majority of *Betaproteobacteria* and in case of *Limnohabitans* usually also its biomass [2]. Just recently, a refined *Limnohabitans* taxonomy has been suggested, based on 35 new isolates retrieved from various standing freshwater habitats and new revised and refined lineages have been proposed [6].

In particular, the R-BT subgroup or subcluster [7] embedded in the genus *Limnohabitans*, which contains at least two species [5], received serious scientific attention enabled by the introduction of a specific FISH (R-BT065) probe targeting this phylogenetically defined group [7]. This included studies aiming at explaining the genus abundance and distribution [8], [9], ecophysiology [3], [6], grazing vulnerability [10], and niche separation [11]. Manipulation experiments [12] suggested that *Limnohabitans* bacteria exhibit an “opportunistic” life strategy. Furthermore, it was suggested that they are able to utilize algal exudates [8], [11]. R-BT cluster bacteria can in general be characterized by large cell volumes, fast growth rates, and pronounced vulnerability to protozoan predation; [2] and references therein.

More than 700 publicly available (GenBank) 16 S rRNA gene sequences of bacteria affiliated with the *Limnohabitans* genus have been retrieved from very different freshwater habitats located on at least three continents. This high number probably suggests a unique role of these bacteria among other ‘typical’ freshwater microbes [1]. Habitats, in which *Limnohabitans* were detected, span from oligotrophic [13] to hypertrophic lakes, include both Arctic [14] and tropical habitats [15], high mountain lakes (Yuhana, unpubl data), lowlands [7], brackish waters [13] and even lower courses of rivers [16], [17]. On the other hand, the natural distribution of *Limnohabitans* bacteria seems to be restricted to running and standing freshwater systems, since reports on detection of this taxon in marine and terrestrial systems are lacking [3]–[6].

The broad spectrum of standing freshwater habitats occupied by *Limnohabitans* bacteria probably suggests an ecological diversification within the *Limnohabitans* genus and accommodation of distinct ecotypes in various ecosystems. Recently [18], such ecological diversification turned out to be the main reason for apparent ubiquity of another important, more narrowly defined, taxon – *Polynucleobacter necessarius* ssp. *asymbioticus* across lentic freshwater habitats. In this previous study, Reverse Line Blot Hybridization (RLBH) probes were used to distinguish 13 groups within the subspecies *P.n. asymbioticus*. The genus *Limnohabitans*, however, represents a much broader group, including probably many more than the currently described four species [6] and is thus expected to show strong intragenus diversification. To address such intriguing question we designed 18 RLBH probes targeting the 16 S–23 S spacer (ITS) region of 35 available isolates of *Limnohabitans* bacteria plus a small number of ITS sequences of environmental clones. By using our set of probes we intended to reveal if the genus wide distribution pattern of single species/species groups is accomplished by broad or narrow adaptations; and moreover if the colonization of a given habitat is executed by a single or multiple *Limnohabitans* groups. A large set of habitats (161 samples) differing in a multitude of parameters, was then screened for presence of probe-defined groups by using the set of newly developed RLBH probes.

The major aim of this study was to provide evidence that the frequent detection of the *Limnohabitans* genus in freshwater habitats results from diversification – random or concerted distribution of different groups based e.g. on biological, physical and chemical parameters. Since the ubiquity of certain bacterial groups [9], [19] is becoming a more and more discussed phenomenon, this manuscript should bring new valuable insights to the current ‘Everything is Everywhere’ debate [18], [20]–[23] and intends to push this dialogue towards new horizons – what is the environmental distribution of the groups within one geographically wide-spread genus? Moreover, the presented study significantly contributed to the knowledge on global biogeographic patterns of bacteria [24] at a higher phylogenetic resolution.

## Materials and Methods

### Sampling of Habitats, Basic Limnological Parameters and *Limnohabitans* Genus Culture Collection

Overall, 161 lentic freshwater habitats (Table S1) were sampled and investigated for the occurrence of Reverse Line Blot Hybridization (RLBH) probe-defined groups of *Limnohabitans* bacteria. Detailed characterizations of the habitats were presented already elsewhere [9], [19]. The habitats represent a large assortment of lakes, ponds, reservoirs and permanent puddles, differing in size, depth, trophic status etc. All habitats are located in Central Europe (Austria and the Czech Republic) and were sampled at depths of 0 to 0.5 m. Assessment of basic limnological parameters such as temperature, oxygen, conductivity, absorbance (as a proxy of DOC concentration) etc., and extraction of environmental DNA were performed as described earlier [19]. Percentage of *Limnohabitans* bacteria (of its R-BT cluster) was analyzed using Catalyzed Reporter Deposition Fluorescence In Situ Hybridization - CARD-FISH [2], [9].

A culture collection comprising 35 isolates [6] was established employing modified filtration-acclimatization method [25] and modified dilution-acclimatization method [26]. Sequencing of the 16 S–23 S rRNA ITS regions of cultivated strains was performed commercially as already described [6]. The sequences of the newly introduced isolates and clones are available [6], submitted under accession numbers HE600660-HE600692.

### *Limnohabitans*-specific PCR Amplification of ITS Sequences and Development of RLBH Probes

*Limnohabitans*-specific amplification of 16 S–23 S ITS sequences from environmental samples was performed by using newly designed primers: *Limnohabitans*-specific forward primer Lim379F (5′-GMAAGYCTGATCCAGCCATT-3′) and the biotinylated (biotin labeled) reverse primer LimCurvITS-R 5′-TTAKTCACTTGACCCTATAACTTTGA-3′ which bind specifically at the beginning and the end of the ITS of *Limnohabitans* bacteria. The conditions of PCR and the specificity of primers were tested in the annealing temperature gradient from 45 to 70°C. Negative controls such as *Polynucleobacter*, *Rhodoferax* and *Polaromonas*, as well as natural bacterioplankton containing and lacking *Limnohabitans* were used to check for the specificity of the primer pair. Moreover, RDP check probe service (http://rdp.cme.msu.edu/probematch/search.jsp) was used to look for the specificity. The length of the PCR product was approximately 1900 bp. The conditions of the PCR reaction were as follows: initial phase at 94°C (3 min), followed by 30 cycles of denaturation at 94°C (1 min), annealing at 65°C (1 min) and extension at 72°C (2 min). The final elongation at 72°C was run for 10 min. In case we obtained low amount of PCR products (lower than ∼50 ng/µl) from some environmental samples, we alternatively performed a nested PCR with universal bacterial ITS primers: forward primer -1406f (5′-TGYACACACCGCCCGT-3′) and reverse primer 23Sr - 5′-GGGTTBCCCCATTCRG-3′ that bind to the 16 S and 23 S rRNA genes, respectively, of all bacteria. The obtained PCR products were subjected to the above described PCR reaction for amplification of *Limnohabitans*-specific ITS sequences. The same PCR conditions were used for both steps of the nested PCR reaction. We were able (in all cases) to amplify the DNA to obtain a sufficient amount (more than ∼ 50 ng/µl) when employing the above nested PCR. *Limnohabitans*-specific RLBH probes were developed, tested and the Reverse Line Blot Hybridization (RLBH) assays were performed according to [18], [27]. In short, biotinylated PCR products were hybridized in a “cross-way” to probes attached in lines on the hybridization membrane. Chemoluminiscence was then used to detect positively hybridized samples.

**Figure 1:**
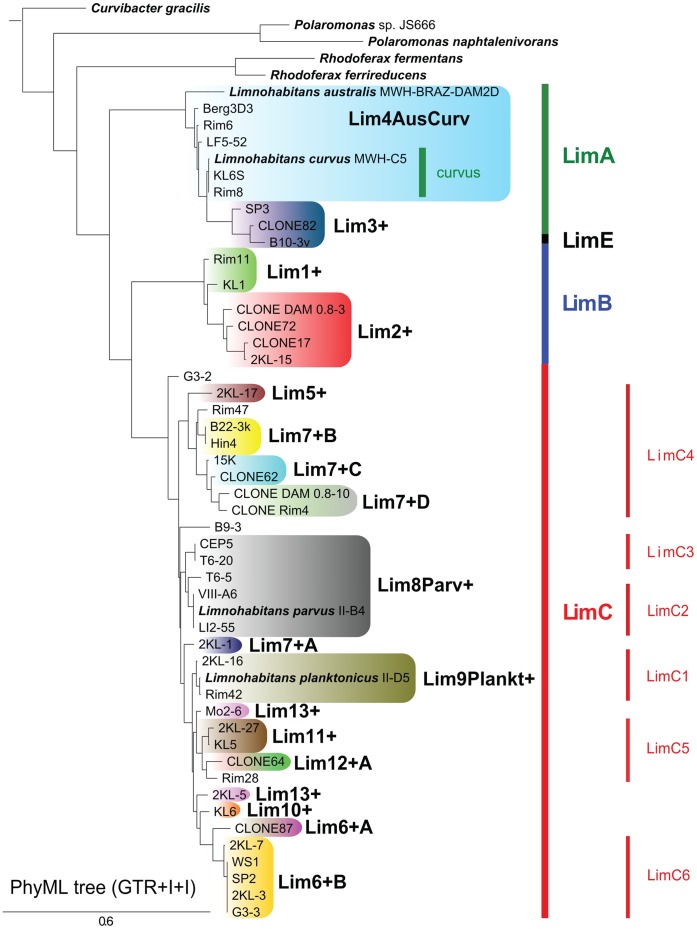
Neighbor-joining tree of 16 S–23 S ITS *Limnohabitans* sequences including probe targets. Neighbor-joining tree based on 16 S–23 S ITS sequences (about 1400 bp) of *Limnohabitans* genus including described species, undescribed strains and environmental clones, following the new *Limnohabitans* phylogeny as suggested by [6]. Groupsgroups targeted by the 18 probes are indicated by different colors. For more information on isolates see [6].

In total, 18 new RLBH probes were designed (for names, sequences and melting temperatures see Table 1, and for their phylogenetic specificity see Fig. 1.) and thoroughly tested (see above) on DNA extracted from 35 available isolates and 9 cloned environmental sequences. All clones used were retrieved from the Římov reservoir in a single sampling campaign, and phylogenetic analyses confirmed clustering of these sequences within the taxon *Limnohabitans*. The tests on specificity of probes were performed in a “cross-way” pattern by using of DNA extracted from various *Limnohabitans* isolates, environmental clones, and natural bacterial assemblages containing and lacking *Limnohabitans* bacteria (see above). Since applied probes differed markedly in signal obtained upon hybridization even when applied to the same amount of target PCR products (from pure cultures), adjustments in concentrations were made to compensate for differences in probe signal intensity [27]. The results were scored as no (0), weak (1), normal (2) and strong (3) as defined in publication [18].

**Table 1:** *Limnohabitans* group-specific probes.

Probe name	Sequence	Tm (°C)
Lim 1+	CTGTGTCAAAGAGTTATTCACATT	55.9
Lim 2+	AAACTTTGTTCGCATTACGGC	55.9
Lim 3+	AAATAGCTTTGATCTTGAAAGAGGT	56.4
Lim 4 AusCurv and Lim 3+	ATTGATTGATTAACTAGGCTGTTC	55.9
Lim 4 AusCurv	AGATATCAGAGTTRCTAGCGG	56.9
Lim 5+	CGGCTGAGGCGTAAGC	56.9
Lim 6+A	ACGACTTGTGCGCATGCT	56.0
Lim 6+B	CGCGTAAATCGAATAAATCCAATA,	55.9
Lim 7+A	CGCAAGCCTCGAGTCATT	56.0
Lim 7+B	CGCAAGCCCAAGTCATTG	56.0
Lim 7+C	CTTATCAAAGGTTTTGATCTCATTC	56.4
Lim 7+D	ACTTATCAAATGTTTTGATCTCATTCAA	56.3
Lim 8 Parv+	TATCGAGTGTTAATRGTGTCTGA	56.2
Lim 9 Plankt+	GGGCCTTGCAGTGGC	56.0
Lim 10+	TTGAGCGGATCCTGCAAG	56.0
Lim 11+	AAGAGATTGCGAGGCTGTTTT	55.9
Lim 12+A	GTTCCCGTAAGGGACTTTAT	55.3
Lim 13+	GGGTCTTGCAAGGGCC	56.9

18 group-specific probes used in the *Limnohabitans* RLBH assays, their 5′ to 3′sequences, and melting temperatures (Tm). Probes target different regions of the 16–23 S rRNA ITS sequences of the *Limnohabitans* bacteria. Note that the probe Lim4AusCurv and Lim3+ combines two probes Lim4 AusCurv and the probe Lim3+.

### Statistical and Graphical Analysis

Principal component analysis (PCA) and Redundancy analysis (RDA) were performed using CANOCO program [28] by including only data on no detection/detection (0/1) of *Limnohabitans* groups by the respective probes (as opposed to other data presentation using the above described scoring by 0, 1, 2, and 3). Environmental parameters were selected using forward selection with 999 Monte Carlo permutations in combination with simple correlation method performed in GraphPad Prism version 5.00 for Windows (GraphPad Software, San Diego California USA, www.graphpad.com). The results of the PCA and RDA analysis done in CANOCO were visualized by CanoDraw for Windows [28].

A potential biogeography of *Limnohabitans* population composition (as assessed by RLBH) was investigated by performing Mantel tests with the software IBDWS (http://ibdws.sdsu.edu/~ibdws/distances.html) following the protocol of [29]. A matrix of pairwise *Limnohabitans* community similarity of habitats was generated by using the software MatGAT (http://www.biomedcentral.com/1471-2105/4/29). A matrix of geographic distance between habitats was generated using the Geographic Distance Matrix Generator (http://biodiversityinformatics.amnh.org/open_source/gdmg/) employing transformed geographic coordinates data. GPS data were transformed from two coordinate GPS values to get one value showing the distance from equatorial in meters by using the software Degrees, Minutes, Seconds to Decimal Degrees calculator freely available at www.satsig.net. In order to test for a potential bias in geographic distribution of habitat types (e.g. biased distribution of acidic or alkaline habitats), Mantel tests with ΔpH data of habitats and geographic distances were performed.

## Results

### *Limnohabitans* Phylogenetic Tree, Probe Targets and Coverage

The set of 18 newly designed RLBH probes (Table 1) covered a vast majority of available isolates and environmental clones (Fig. 1). In total, we managed to target and detect approximately 92% of isolates and clones currently represented by ITS sequences. The designed probes target sequences ranging from single isolates, e.g. probe Lim7+A detecting the isolate 2-KL1, and Lim12+A detecting the environmental CLONE87, respectively, to probes that detect up to seven different genotypes (Fig. 1), such as the probe Lim4AusCurv that was designed based on isolates retrieved from various countries such as Brazil, France, and the Czech Republic. On average, each RLBH probe covered in total 2.5 isolates, type strains of described species or clones.

LimA lineage genotypes [6] were targeted by the Lim4AusCurv probe (and also Lim4AusCurv and Lim3+ joint probe, see below). LimE lineage (represented by the sole isolate B103v) was covered (together with other isolate and one clone) by the Lim3+ probe. LimB lineage was a target for the probes Lim1+ and Lim2+A. A wide LimC lineage (and its subgroups LimC1 to LimC6) was targeted by a wide array of probes (Lim5+, Lim6+A, Lim6+B, Lim7+A, Lim7+B, Lim7+C, Lim7+D, Lim10+, Lim11+, Lim12+A, Lim13+, and Lim8Parv+) covering isolates originating from various habitats.

Probes with names followed by+(e.g. Lim1+) target isolates and/or clones possessing the discriminative sequence characteristic for the R-BT065 bacteria (hybridizing with the R-BT065 FISH probe). The group targeted by the probe Lim4AusCurv, includes among others *Limnohabitans australis* and *L. curvus,* is not targeted by the R-BT065 FISH probe. Thus results obtained by this probe represent the first report on distribution of the targeted taxon in environmental samples so far.

### Relative Abundance of the Probe-defined Groups of the *Limnohabitans* Genus, Trends in the Distribution of the Groups Across Habitats, and Niche Separation Among the Groups

RLBH signals from 18 probes applied to samples of 161 habitats were scored as negative (0), weak (1), normal (2), and strong (3), corresponding to grayscale depictions in the overview of the raw data presented in the Table S1. These raw data represent an average from triplicated measurements. The intensity of the signals score were related to the strength of the signals obtained when testing the individual probes against available isolates and clones. This rule had to be applied when testing the RLBH probes since the probes returned signals of markedly different strength.

**Figure 2:**
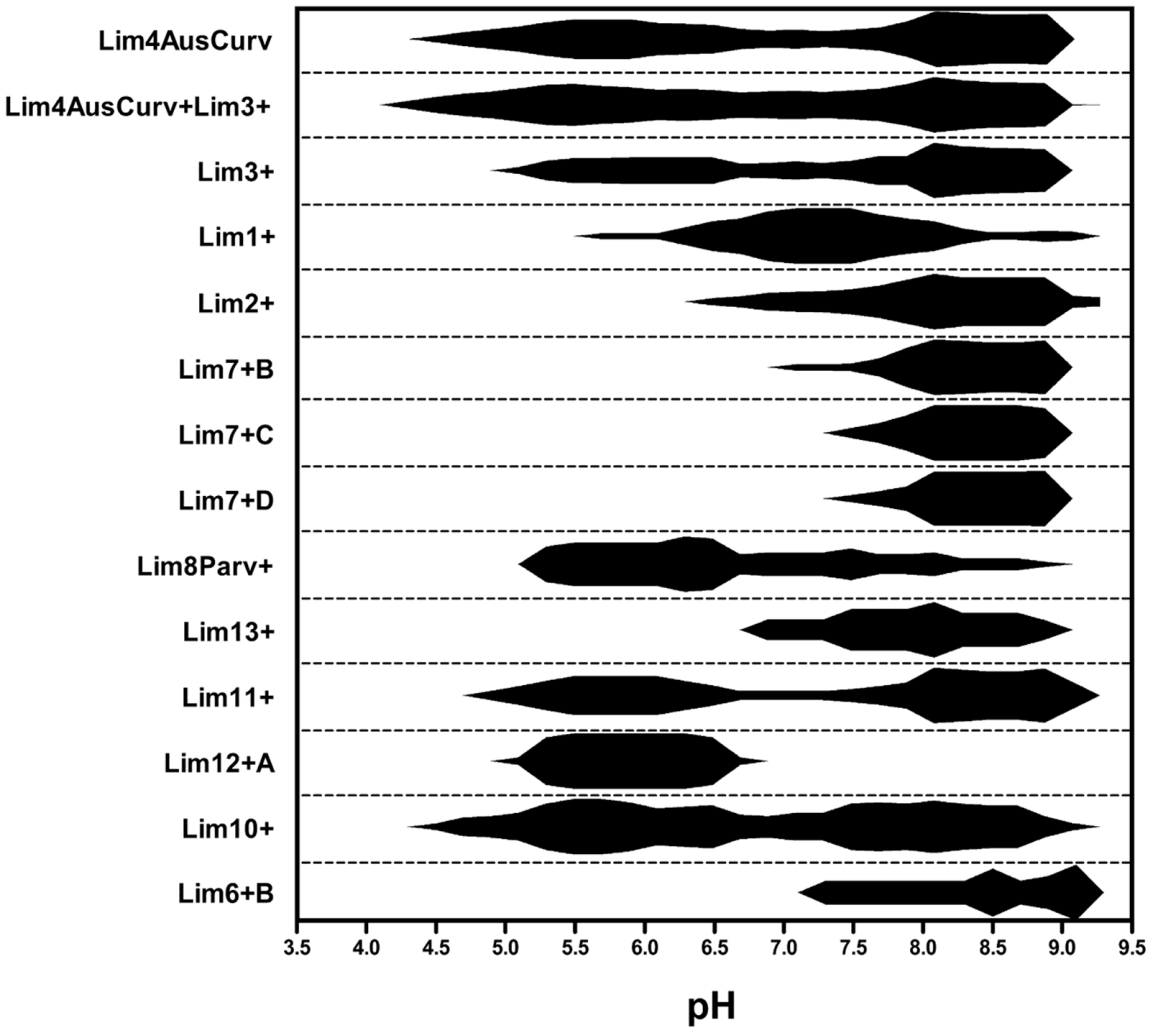
Proportions of different *Limnohabitans* probe-defined groups along the increasing pH gradient. Relative proportions of different *Limnohabitans* genotype groups along the increasing pH gradient of 161 habitats. The pattern was calculated as follows: Each signal has been scored as either 0 (no signal) or 1 or 2 or 3 (depending on strength), pH scale has been categorized to make classes of 0.2, for each *Limnohabitans* group the score has been summed up within the 0.2 pH class. The intensities of occurrence found in each pH class (0.2) were (for each group) expressed as a fraction of the total score in all habitats. The sum of all generated intensities of occurrence was thus for each group equal to 1 within each pH class (0.2). The relative intensities were expressed for each *Limnohabitans* group as a ratio between the actual and the maximal found normalized relative intensity. As a result – normalized relative intensities have values from 0 to 1. Gliding average was than calculated averaging 7 values (middle ±3 = 7). Program used: GraphPad Prism v. 5.

Very contrasting trends were revealed when comparing an overview of the relative distribution of the *Limnohabitans* groups along a pH gradient of the sampled habitats (Fig. 2) ranging from pH 3.8 to 9.0. In Fig. 2 only detection (scoring categories 1, 2, 3) and lack of detection (scoring category 0) by a given probe is presented. Some of the 18 designed and employed RLBH probes did not return any or returned only very weak and unreliable detection signals when applied to the sample collection. These probes were: Lim5+, Lim6+A, Lim 7+A, and Lim9Plankt+, and results of these probes were therefore excluded from Fig. 2. These probes mainly target isolates from the Klíčava reservoir [6], isolates from the Římov reservoir, the *L. planktonicus* type strain, and two environmental clones.

Taxa targeted by the probes Lim7+B, Lim7+C, Lim7+D, respectively, were mainly found in a narrow range of habitats (Fig. 2), ranging from pH 7.5 to 9.0. A similar, but slightly wider pH range was occupied by taxa detected by probes Lim2+ and Lim13+, respectively.

Some groups showed a bimodal (two-peak) distribution along the pH gradient of investigated habitats – Lim4AusCurv, Lim4AusCurv+Lim3+, Lim3+, Lim11+, respectively, and to a certain extent also the genotypes targeted by Lim10+. *Limnohabitans* groups targeted by the probe Lim1+ showed a narrow range of detection only in circum-neutral habitats (about pH 7), showing however strong signals, and being only rarely detected in habitats of pH higher than 8.2.

Probe Lim8Parv+ targeted genotypes (based on isolates retrieved from the Římov reservoir, Nový u Cepu pond, Lake Loosdrecht in Netherlands, etc.) exhibited a preferred range of pH 5 to 6 followed by a gradual decrease in the strength of their signal towards pH of 9.

Genotypes targeted by probe Lim12+A represent the sole group of *Limnohabitans* bacteria strongly preferring an acidic range of habitats, being virtually absent in habitats of pH higher than 6.8. They also exhibited the narrowest range of habitats occupied.

**Figure 3:**
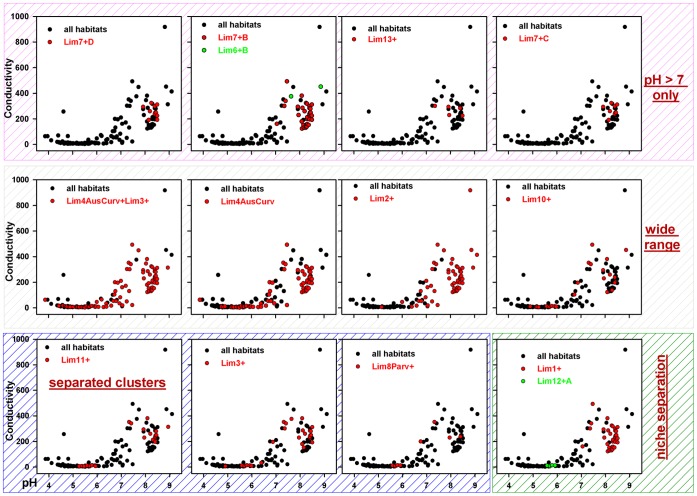
Probe-defined groups and their relationship to conductivity and pH values. Selected probe-defined *Limnohabitans* groups and their relationship to conductivity vs. pH values suggesting possible scenarios of ecological distribution of given *Limnohabitans* groupsin standing freshwater habitats located in Europegroup.

A complete niche separation of *Limnohabitans* groups targeted by the Lim12+A probe from some other groups (Lim6+B, Lim7+B, +C, +D, Lim13+) was evidenced (Fig. 3).

Based on the data presented, and the relationship of *Limnohabitans* groups to pH and conductivity (Fig. 3) we proposed a separation of *Limnohabitans* groups and their clustering to several categories – broad distribution without pH preferences, preference for alkaline or acidic habitats, or two-peak distribution.

### Factors Influencing the Presence of Distinct *Limnohabitans* Groups

**Figure 4:**
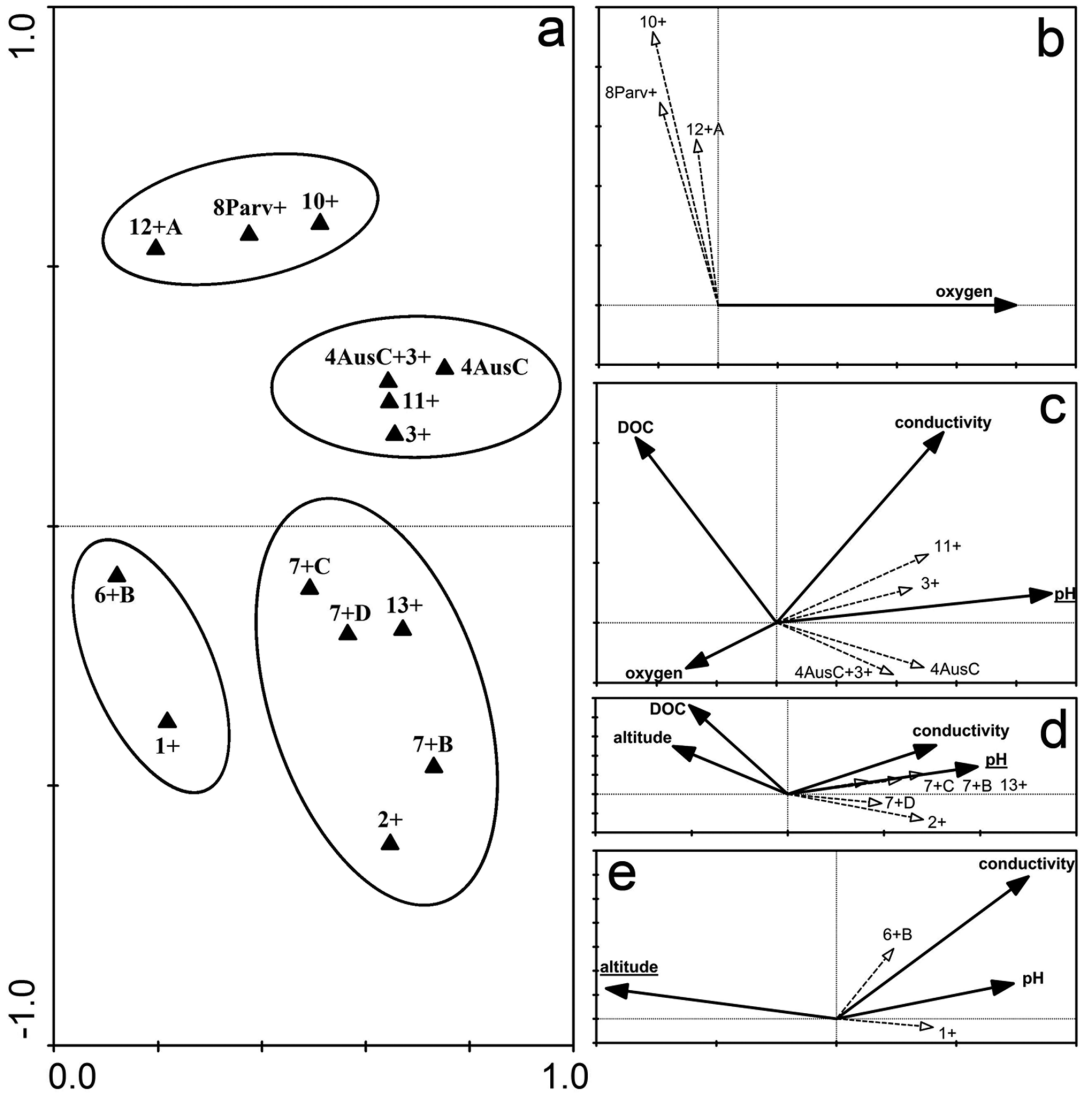
Occurrence similarity clusters of the *Limnohabitans* probe-defined groups. Occurrence similarity clusters of the *Limnohabitans* probe-defined groups; panel a: Principal component analysis (PCA) of all groups, 53.2% of the explained variability. Panels: b, c, d, e –Redundancy analysis (RDA) for distinct clusters presenting different environmental parameters responsible for the group occurrence in the environment. Underlined parameters have the strongest influence on the respective *Limnohabitans* groupsgroup. All probe names have been shortened by excluding ‘Lim’ in the name of the probe.

Based on similar occurrence pattern in the environment, we could divide *Limnohabitans* probe-defined groups into four ‘environmentally similar’ clusters (Fig. 4b–e) by using Principal Component Analysis (PCA) - Fig. 4a. Cluster I (Fig. 4b) combines groups Lim8Parv+, Lim10+ and Lim12+A; all of them strongly correlated with oxygen concentration only (negative correlation, p<0.001). Cluster II (Fig. 4c) contains groups Lim11+, Lim3+, Lim4AusCurv and Lim4AusCurv+Lim3+ and its distribution along the habitat range was impacted by pH and conductivity (positive correlation, p<0.001) and DOC and oxygen (negative correlation, p<0.01). Cluster III (Fig. 4d) includes groups Lim2+, Lim7+B, Lim7+C, Lim7+D and Lim13+. pH had the strongest impact on the distribution of these (positive correlation, p<0.001), and all other parameters (conductivity, DOC and altitude), which were correlated with pH, seemed to be of lower importance. Cluster IV (Fig. 4e) joins together groups Lim6+B and Lim1+ which fell apart from other genotypes. Environmental parameters with effect on these groups were pH and conductivity (positive correlation, p<0.001) together with altitude, which was discovered to have the strongest influence (negative correlation, p<0.001).

### *Limnohabitans* Group Richness Along the pH Gradient

**Figure 5:**
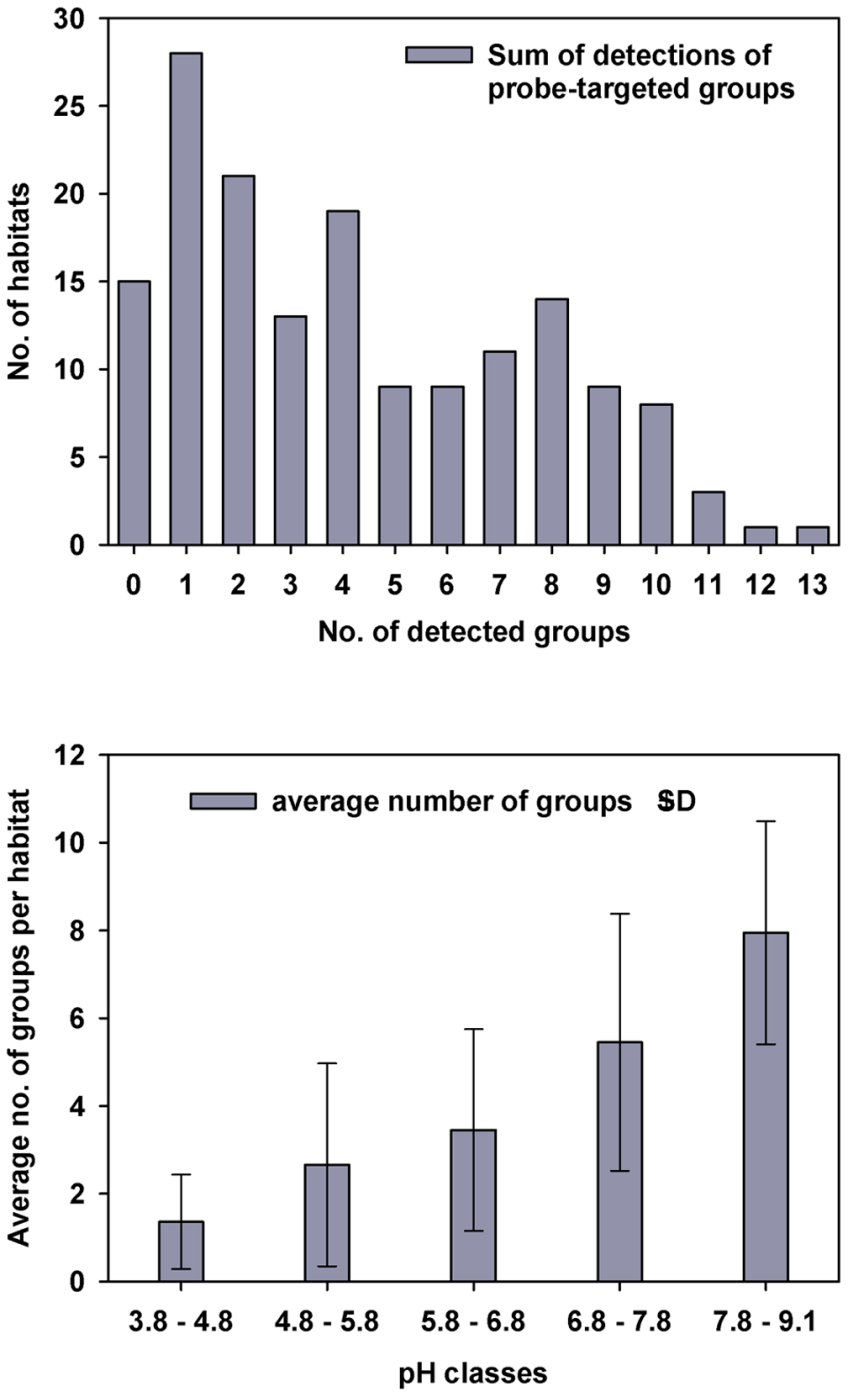
Overview statistics on Detected *Limnohabitans* groups in 161 investigated freshwater habitats. Upper panel: Sum of detected *Limnohabitans* groups in a given number of habitats, e.g. 0 groups were detected in 15 habitats, sum of 2 groups in 22 habitats, 5 groups coexisting in 9 habitats etc. Lower panel: average number of groups detected per habitat across habitats grouped in ‘pH classes’ of approximately 1. SD – Standard Deviation.

Samples from 15 habitats (9.3% of the total) resulted in a complete lack of detection by any of the 18 RLBH probes. Detection by only a single probe was recorded in 28 (17.4%) habitats, 21 (13%) habitats were inhabited by two *Limnohabitans* groups, 8% by three groups, and 11.8% by four groups. There was an evident trend (Fig. 5, upper panel) of a gradual decrease of number of habitats with a higher number of probe-detected groups inherent –11, 12 and 13 concurrent groups were found in 3, 1 and 1 habitat only, respectively.

An increasing trend in amount of probe-defined groups with increasing pH of investigated habitats was observed (Fig. 5, lower panel). On average, alkaline habitats showed more than 2.5 times higher detection frequencies of *Limnohabitans* groups than habitats characterized by low pH – on average 2.6 groups at pH range 3.8 to 7 contrary to 7.4 groups at pH range 7 to 9.1. A marked difference between groups of pH 3.8–4.8 and the group above pH 7.8 was observed.

### Mantel Test on Biogeography of *Limnohabitans* Assemblages

*Limnohabitans* assemblage (expressed as similarity values of RLBH detected probe-defined groups) was found to correlate negatively, but only weakly, with geographic distances of habitats (r = - 0.1117, p<0.0200, 10 000 randomizations). However, pH as a main driver of *Limnohabitans* population composition (see above) also correlates with geographic distance (r = −0.0819, p<0.0200, 10 000 randomizations), suggesting that the influence of distribution of *Limnohabitans* assemblages was influenced by geographic distribution of habitat types.

## Discussion

### Distribution of ITS-defined Groups of *Limnohabitans*

The major aims of this study were to test for ecological diversification within the *Limnohabitans* genus and to test for niche partitioning among *Limnohabitans* groups by revealing their specific distribution patterns along environmental gradients (e.g. pH of habitats).

Different scenarios have been encountered when analyzing the distribution of taxa showing a clear preference of distinct groups for either alkaline or acidic habitats, however, the majority of groups could be detected in alkaline as well as acidic waters. In some cases, ‘bimodal’ distribution (e.g. Lim4AusCurv, Lim11+ etc.) was observed suggesting more profound ecological diversification within the group and probably different ecological adaptations of the distinct members within the given group. Even a complete niche separation between some groups could be suggested when comparing genotypes hybridizing, for instance, with the probe Lim12+A and Lim6+B.

Out of the total of 18 newly designed RLBH probes, 14 have returned recordable signals across the 161 habitats investigated (cf. Fig. 2). The remaining probes displayed either no hits **–** Lim5+ and Lim9Plankt+ **–** or few extremely weak signals **–** probes Lim6+A and 7+A.

Interestingly, we have revealed a probable clear trend of replacement of one group with another along the increasing pH gradient. Groups covered by the Lim1+ and Lim2+ probes are such examples (cf. Fig. 2), displaying strong signals in most cases, restricted to a relatively narrow pH range, probably suggesting adaptation to a rather narrow range of ecological conditions. Importantly, to our current knowledge, probes Lim1+ and Lim2+ completely cover group LimB (Fig. 1, [6]), which harbors the majority of partial 16S rRNA sequences of *Limnohabitans* bacteria (in total more than 700) currently available in GenBank.

Unfortunately, no isolates originating from low pH habitats were retrieved so far [6], making it impossible to design appropriate probes. We are fully aware of a significant drawback brought about by this fact, although the low pH and humic substances rich habitats are generally poor in *Limnohabitans* bacteria [2]. We believe that the lack of these probes should not be overemphasized, considering the major pH range, inhabited by *Limnohabitans* bacteria [9]. Moreover, one of the probe-defined groups of bacteria – targeted by the Lim12+A probe – was found solely in low pH habitats. This probe was however designed based on a single clone sequence originating from a circum-neutral habitat.

In this study we also provided the very first insights into the distribution and ecology of *L. australis*, *L. curvus*, and other isolates originating from Brazil, Czech Republic, Austria and France, targeted by the Lim4AusCurv probe. These microbes do not represent the ‘true R-BT bacteria’, and are not targeted by the original R-BT065 FISH probe [7]. So far, they were thus almost completely excluded from the analysis on distribution of *Limnohabitans* bacteria with only one exception, i.e. application of relatively broad and undefined, 16 S rRNA targeted probe Rho-BAL47–396 designed by Zwart and colleagues [27], which was previously used to cover a ‘cluster-like’ group. Bacteria targeted by the Lim4AusCurv probe showed a two-peak distribution with one maximum around pH of 5.5 and the other around 8.5. Signals recorded from this group were always very strong and distributed over a wide pH range, suggesting a significant contribution of Lim4AusCurv genotypes to the environmental distribution of *Limnohabitans* bacteria. Based on these results, we may speculate that if the “non-R-BT” *Limnohabitans* bacteria contribute significantly to *Limnohabitans* populations in a broad variety of habitats, the relative abundances of *Limnohabitans* presented in recent studies using the R-BT065 FISH probe [2], [9] might have been significantly underestimated. In this case, a development of new FISH probes for precise quantification of other *Limnohabitans* groups is highly desirable in order to complete the whole picture of *Limnohabitans* distribution.

As opposed to the widely distributed group Lim4AusCurv (*L. australis* and *curvus*), group Lim9Plankt+, targeting among others also *L. planktonicus*, was not detected in our set of 161 habitats. *L. planktonicus*, isolated from the Římov reservoir [5], has just recently been used for laboratory experiments [11], suggesting its probable niche separation from other closely related species. Together with *L. parvus* [5] – targeted by Lim8Parv+ RLBH probe, they currently represent the only two described species originating from this canyon-shaped reservoir. Contrary to Lim9Plankt+ genotypes, Lim8Parv+ *Limnohabitans* groups were found occupying a wide range of habitats, spanning from pH 5 to 9. The distribution pattern they showed (cf. Fig. 2) looked however very different from other groups, forming a maxima around pH 5.5 to 6.5 and a markedly decreasing trend towards higher pH values.

We demonstrated for the first time a clear trend in lower diversity of *Limnohabitans* groups in the acidic part of the habitat spectrum as opposed to the alkaline part. Some habitats with very low pH (3.8–4.5) even completely lacked any signal from the RLBH probes (RLBH based, specific PCR almost always returned a measurable product), confirming low abundance of *Limnohabitans* bacteria in pH low (3.8–4.5) habitats as previously demonstrated [9]. From this point of view, the microdiversity of *Limnohabitans* bacteria in low pH habitats can be expected to be rather low, but the true picture still remains unresolved.

### *Limnohabitans* Genus Diversity and Distribution

Studying *in situ* diversity of a given group of microbes is indeed problematic and providing a comprehensive study of any type of biological diversity would be complicated beyond feasibility if nearly every individual organism were ecologically unique [30].

Based on the so far gathered data, we may generally assume a high diversity as well as dispersal potential for *Limnohabitans* genus bacteria since it is virtually omnipresent in standing freshwater habitats as already evidenced earlier [9].

We revealed, for most of the investigated *Limnohabitans* taxa, neither random nor even ubiquitous distribution pattern across 161 habitats sampled. However, in a number of cases we identified *Limnohabitans* groups showing bimodal distribution – i.e. displaying two maxima of occurrence (cf. Fig. 2). These groups probably do not constitute ecologically coherent assemblages of strains or ecotypes with generalist adaptations, but ecologically heterogeneous groups consisting of two or more groups with distinct ecological adaptations.

Moreover, some of the probe-defined groups showed extremely broad distribution patterns (genotypes targeted by the probe Lim 10+, Lim4AusCurv etc.) stretching from really low pH (3.8–4.5) to alkaline (8.0–8.5) habitats - an elucidation for this broad detection may also be a deficiency in specificity of probes, i.e. match with *Limnohabitans* groups not included in the culture collection and consequently not tested in empirical probe validation.

The results of our study do not support the hypothesis that the high percentage of habitats containing *Limnohabitans* genus across a large set of different standing freshwater systems results from a generalist adaptation of these bacteria. Several of the *Limnhabitans* groups showed clear habitat preferences and restricted ecological distributions and their composition can partially be explained by a metapopulation theory [31], [32] claiming that a metapopulation comprises a group of spatially separated populations of the same species that interact at some level. Distinct ecotypes are subsequently selected from a local ‚metapopulation‘ adapted best to the environmental conditions – a so called ‘ecotype sorting‘. Probable causes of such ecotype sorting are environmental drivers as suggested in this study as well as previously [18].

### What Level of Phylogenetic Resolution is Required to Understand the Ecological Function of *Limnohabitans* spp.?

BetI lineage [1] of *Betaproteobacteria* that includes the *Limnohabitans* genus is currently divided, according to Newton and colleagues [1], into two clades, i.e. betI-A and betI-B, which are synonymous with the genera *Limnohabitans* and *Rhodoferax*, respectively [1]. BetI-A clade can further be split [1] into four tribes – Lhab-A1 to A4, first two of them being targeted by the R-BT065 FISH probe. Just recently, a subdivision of BetI-A clade into many subgroups was proposed [6], based on a significant number of newly available isolates and introduced LimA, B, C, D, E and H lineages; and LimC1-LimC6 as groups of LimC lineage (compare Fig. 1 and [6] reference).

The division into 4 major groups (based on 16 S rRNA gene phylogenies) made by Newton turned out to be inefficient for distinguish subgroups of *Limnohabitans* genus under natural conditions in the scope of our study, that is why we used the better resolving, i.e. 16 S–23 S rRNA ITS sequences based, classification proposed by Kasalický [6] for the design of a set of RLBH probes (Fig. 1). Based on the results gained here, showing different patterns (such as two-peak distribution, niche separation etc.) even within the Lhab tribes defined and suggested by [1], we believe that a more appropriate system is represented by the one suggested by Kasalický et al. [6] since the subdivision of betI-A clade in four taxa proposed by [1] results in ecologically heterogeneous rather than coherent ecological groups. Moreover, 16 S rRNA and ITS sequences of numerous new isolates as well as their physiology and morphology is known, representing thus more detailed and appropriate system for assessing diversity.

The LimA lineage ([6], also known as Lhab-A3 [1]), including among others *L. curvus* and *L. australis,* shows a within-lineage sequence similarity of >98% on 16 S rRNA genes and >89% on ITS sequences and was covered completely in our study by the Lim4AusCurv and Lim3+ probes, as well as by a combined probe Lim4AusCurv+Lim3+ targeting both groups (Fig. 1).

The LimB lineage (16 S rRNA similarity >99.5%, ITS similarity >89.9%), was covered completely by Lim1+ and Lim2+ probes (Fig. 2). A clear ‘replacement’ of one probe-defined group of *Limnohabitans* by the other (Fig. 2) along the pH gradient was observed.

A large group of the LimC lineage (including *L. planktonicus* and *L. parvus* and other 25 newly isolated strains, [6]) share rather high similarities of their 16 S rRNA genes (>98.4%) and of their ITS sequences (>89%). The whole LimC lineage was in this study almost completely covered (apart from three isolates) by our set of RLBH probes – LimC4 group was targeted by Lim5+ (not detected across 161 habitats investigated) and Lim7+B, C and D – all showing rather restricted range of occurrence. LimC2 and LimC3 groups were targeted by the Lim8Parv+ probe. Bacteria targeted by the Lim9Plankt+ (group C1) were surprisingly not found across our habitat set, pointing at possible underestimation or unspecificity of a previously successfully tested probe. LimC5 group (covered by Lim11+ and Lim12+A), although being very similar in their ITS sequence, showed contrasting trends: Lim11+ displaying a classical bimodal distribution, contrary to Lim12+A targeted bacteria found mostly in acidic habitats. In this special case (LimC5), diversity brought about by ecological drivers could have been more pronounced (in terms of observed distribution patterns) than what one would deduce based only on the sequence similarities of both groups. Lim13+ and Lim10+ probes, currently assigned only to the large LimC group showed contrasting trends (Fig. 2). The morphologically exceptional subgroup LimC6 (targeted by the probe Lim6+B), characterized by largest mean cell volume, showed rather restricted distribution pattern (occupying a narrow pH range of habitats) and returning “weak“ signals (mostly scoring category 1) from the natural samples.

### Conclusions

In continuity to the recently published work by Newton and colleagues [1], we have to point out that our study offered higher phylogenetic resolution (ITS versus 16 S rRNA), thus providing deeper insights into the problematics of the *Limnohabitans* genus delineation. Deducing from the results we can also claim we gained first insights that might help in distinguishing LimA lineage (Lhab-A1) and LimB and LimC lineages (Lhab-A2). As proposed by [6], an employment of fine-resolution genetic markers such as the ITS region as opposed to the traditional 16 S rRNA gene sequence, turned out to be very fruitful. However, we are surely aware of the fact that in some cases – bimodal distribution for several probe-targeted groups, large distribution pattern observed, etc., the resolution provided by the employed RLBH probes is not high enough and even higher resolution (mainly employing the qPCR method) is required if we want to understand the ecology of the *Limnohabitans* genus properly. Nonetheless, higher resolutions and larger inventory studies of habitats representing broad ecological and geographic gradients are required in order to better understand the ecology and biogeography of freshwater bacterioplankton taxa.

However, our approach represents a very first and thus crucial improvement in following the resolution of the *Limnohabitans* genus microdiversity under natural conditions since the introduction of the fluorescence R-BT065 FISH probe only specific for the R-BT cluster more than 10 years ago.

## Supporting Information

Table S1 RLBH detections of probe-defined groups scored as no (no color), weak (light grey color), normal (dark grey color) and strong (black color) across all 161 habitats. For more parameters of habitats see Jezberová et al., 2010; Šimek et al., 2010; Jezbera et al., 2011 and 2012.
(DOC)
