# Supplementary material for: Patterns of Limnohabitans Microdiversity across a Large Set of Freshwater Habitats as Revealed by Reverse Line Blot Hybridization
Source: PLoS One. 2013 Mar 12;8(3):e58527. doi: 10.1371/journal.pone.0058527 (PMC3595293; doi:10.1371/journal.pone.0058527)
Supplement: Table S1 — RLBH detections of probe-defined groups scored as no (no color), weak (light grey color), normal (dark grey color) and strong (black color) across all 161 habitats. For more parameters of habitats see Jezberová et al., 2010; Šimek et al., 2010; Jezbera et al., 2011 and 2012. (DOC) [file pone.0058527.s001.doc]

**Table S1.** RLBH detections of probe-defined groups scored as no (no color), weak (light grey color), normal (dark grey color) and strong (black color) across all 161 habitats. For more parameters of habitats see Jezberová et al., 2010; Šimek et al., 2010; Jezbera et al., 2011 and 2012.
